# Hypertension worsens bone loss and weakens bone in aged ovariectomized mice

**DOI:** 10.1093/jbmrpl/ziag101

**Published:** 2026-06-15

**Authors:** Elizabeth M Hennen, Sasidhar Uppuganti, Santosh Thapa, Néstor de la Visitación, Priya Mathiy, Gabriel Ramirez, Elizabeth Rendina-Ruedy, David G Harrison, Jeffry S Nyman

**Affiliations:** Department of Biomedical Engineering, Vanderbilt University, Nashville, TN 37232, United States; Department of Orthopaedic Surgery, Vanderbilt University Medical Center, Nashville, TN 37232, United States; Department of Medicine, Vanderbilt University Medical Center, Nashville, TN 37232, United States; Department of Medicine, Vanderbilt University Medical Center, Nashville, TN 37232, United States; Department of Health Humanities, Pennsylvania State University, Abington, PA 19001, United States; Department of Cell and Developmental Biology, Vanderbilt University School of Medicine, Nashville, TN 37232, United States; Department of Medicine, Vanderbilt University Medical Center, Nashville, TN 37232, United States; Department of Nutritional Sciences, College of Allied Health, University of Oklahoma Health Campus, Oklahoma City, OK 73117, United States; Department of Medicine, Vanderbilt University Medical Center, Nashville, TN 37232, United States; Department of Biomedical Engineering, Vanderbilt University, Nashville, TN 37232, United States; Department of Orthopaedic Surgery, Vanderbilt University Medical Center, Nashville, TN 37232, United States; United States Department of Veterans Affairs, Tennessee Valley Healthcare System, Nashville, TN 37212, United States

**Keywords:** hypertension, menopause, bone strength, trabecular architecture, cortical structure

## Abstract

The occurrence of both hypertension and osteoporosis increases after menopause, and fracture risk is higher in adults with hypertension than in adults without this chronic disease. We hypothesized that ovariectomy (OVX)-induced estrogen-deficiency and angiotensin (Ang) II-hypertension together cause a greater decline in bone strength than OVX alone. Ovaries were removed (OVX) or remained intact (Sham) in 12-mo-old, C57BL/6J mice. Four weeks after surgery, mice received either an infusion of vehicle or angiotensin II at 490 ng/kg/min for 6 wk to induce hypertension. We monitored blood pressure (BP) and areal BMD (aBMD) before surgery, before infusion, and before euthanasia. OVX augmented the increase in BP in hypertensive mice. OVX, not hypertension, decreased whole-body aBMD. Nonetheless, hypertension lowered cortical bone area and thickness of the femur mid-diaphysis in only the OVX group. As determined by the ultimate force that the femur mid-diaphysis endured during 3-point bending, OVX lowered the strength of cortical bone when mice were hypertensive; and hypertension did the same when mice were estrogen deficient. Hypertension and OVX independently affected trabecular bone, such that reductions in trabecular thickness and tissue mineral density were additive with the lowest mean observed in the OVX-hypertension group. OVX significantly lowered the compressive ultimate force of the lumbar vertebra when controlling for hypertension group. Although hypertension did not significantly affect vertebral strength, OVX-hypertension significantly reduced this parameter. The addition of hypertension to OVX in 12-mo-old female mice weakened bone beyond either effect alone. Thus, hypertension, which commonly occurs after menopause or ovarian loss, increases the likelihood of osteoporosis, and thus indicates a high-risk population.

## Graphical Abstract

**Graphical Abstract:**
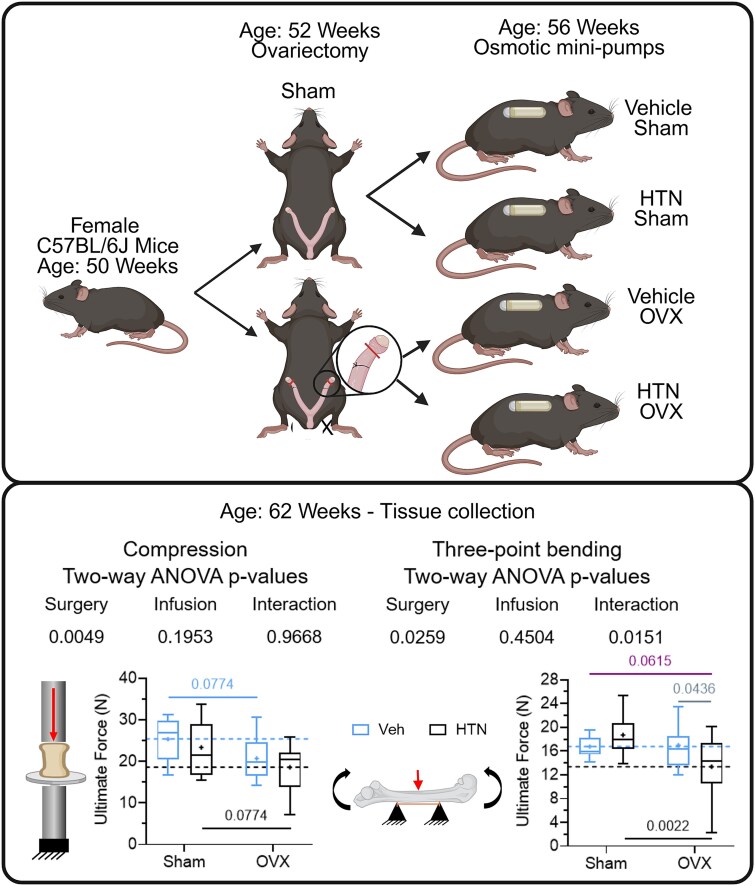

## Introduction

The age-related decline in estrogen is well known to reduce bone mass and increase the occurrence of fragility fractures.^1^ Additionally, post-menopausal women are 3-to-4 times more likely to suffer a fragility fracture than age-matched men.^2^ The risk of hypertension also significantly increases during menopause.^3^ Like osteoporosis, hypertension is more prevalent in postmenopausal women than age-matched men.^4^^,^^5^ Hypertension is the leading modifiable risk factor for cardiovascular diseases with high morbidity and mortality.^6^^,^^7^ Likewise, low-energy fragility fractures increase the risk of death, and patients with a hip fracture have a much higher mortality risk when they have a comorbidity like hypertension.^8^ Thus, treating both osteoporosis and hypertension are clearly beneficial, but the causal relationship between the 2 chronic diseases is poorly understood.

There is a clinical association between osteoporosis and hypertension. Areal BMD (aBMD) is lower, while the risk of a fragility fracture is higher in hypertensive adults than in age- and sex-matched adults with normal blood pressure (BP).^9^^,^^10^ In postmenopausal Caucasian women, the rate of bone loss over 3.5 yr was 0.35% per year in the lowest quartile of BP and 0.70% per year in the highest quartile^11^; and an inverse relationship between BP and BMC per body mass was observed in 39 Hispanic women (5 postmenopausal).^12^ In addition, the odds ratio for hypertension (systolic BP ≥ 140 mmHg and/or diastolic BP ≥ 90 mmHg or intake of antihypertensive drugs) among Chinese postmenopausal women having osteoporosis was 1.832 (95% CI, 1.495-2.246).^13^ Treating hypertension with certain BP-lowering medications like angiotensin converting enzyme (ACE) inhibitors or angiotensin receptor blockers (ARBs) lower the risk for fragility fractures.^14^ It is unknown whether such drugs lower fracture risk by lowering BP or by direct actions on bone metabolism.

Ovariectomy (OVX) and continuous infusion of angiotensin II (Ang II) are two widely used preclinical, animal models to investigate treatment strategies for postmenopausal osteoporosis and hypertension, respectively. Both models have classically relied on young mice (eg, 3-6 mo of age) to observe significant changes in bone mass and BP.^15–20^ Compared to intact, age-matched C57BL/6J mice, mice that underwent OVX surgery 25 d before Ang II infusion (800 ng/min/kg body weight) had a greater increase in mean arterial pressure.^21^ A follow-up study using the same model of hypertension found that genetic deletion of the estrogen receptor-alpha (ERα^−/−^) also increased mean arterial pressure.^22^ Further supporting the protective role of estrogen signaling, treating 16- to 18-mo-old female mice with the specific estrogen compound 17β-estradiol (E_2_) lowers BP following the administration of Ang II (600 ng/min/kg) to levels observed in 3- to 4-mo-old female mice.^23^ Thus, beyond causing bone loss, OVX or menopause also abrogates the effect of estrogen to counteract Ang II-related vasoconstriction and retention of water.^24^

With respect to bone in female mice, the effect of hypertension has only been investigated in C57BL/6J mice after 12 wk of estrogen deficiency due to OVX surgery (age not specified); and, hypertension, specifically Ang II at 200 ng/min/kg for 4 wk, reduced trabecular bone volume fraction of the tibia metaphysis when compared to OVX mice that did not receive Ang II.^25^ It is unknown whether hypertension weakens bone in intact female mice like it does in intact male mice^26^ or requires OVX. In 3- to 4-mo-old male mice, causing hypertension in either the Ang II model (490 ng/min/kg) or the deoxycorticosterone acetate (DOCA)-salt model (low circulating Ang II) significantly reduced bone mass and bone strength.^26^

Working toward a clinically relevant mouse model of hypertension and osteoporosis, we hypothesized that hypertension and OVX-estrogen deficiency together worsen the decline in bone mass and bone strength than either infusion or surgery alone. To test this hypothesis, we evaluated the effect of ovariectomy, hypertension, or their combination on the fracture resistance of bone in 12-mo-old mice because this age in C57BL/6J mice mimics the reproductive age of women approaching menopause.^27–29^ To replicate the typical prognosis of hypertension in women, hypertension was induced after estrogen loss.^30^ By establishing how estrogen deficiency and hypertension affect bone, effective treatment strategies to prevent fractures in postmenopausal, hypertensive women can possibly be investigated in a preclinical model.

## Materials and methods

### Study design

**Figure 1:**
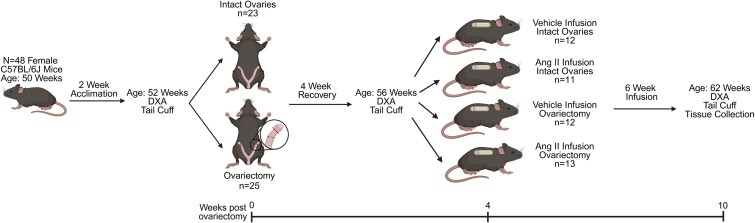
Study design. Sham and ovariectomy surgery were performed at 52 wk of age. Osmotic minipumps were implanted at 56 wk of age to continuously infuse vehicle or angiotensin II. Areal BMD (DXA) and blood pressure (tail cuff) were longitudinally measured. Mice were euthanized at 62 wk of age. Figure made in Biorender.

Female C57BL/6J mice at 50 wk of age from Jax (The Jackson Laboratory) were randomly assigned to 4 groups: (1) sham surgery and vehicle infusion, (2) sham surgery and Ang II infusion, (3) ovariectomy surgery (OVX) and vehicle infusion, and (4) OVX and Ang II infusion (Figure 1). The mice acclimated for 2 wk before surgery. At 52 wk of age, mice underwent sham surgery or bilateral ovariectomy under anesthesia (inhalant isoflurane). Four weeks later, at 56 wk of age, Ang II (490 ng/min/kg of body weight) or vehicle infusion was administered by subcutaneous osmotic mini-pumps (Catalog #: 2006, Alzet) for 6 wk.^26^ Typically, the BP is elevated by the third or fourth day of Ang II infusion and gradually increases up to 10 d after infusion.^19^^,^^31^ Thereafter, the BP stabilizes for the remaining period of infusion. Mice were weighed weekly until euthanasia at 62 wk of age by CO_2_ inhalation before tissue collection. Uteri from all mice were weighed to determine if ovariectomy was successful. One mouse was excluded due to failed ovariectomy (normal uterus weight). All experimental procedures were approved by the Vanderbilt University IACUC and were conducted in an American Association for the Accreditation of Laboratory Animal Care-accredited facility in accordance with the Guide for the Care and Use of Laboratory Animals and the Public Health Service Policy on Human Care and Use of Laboratory Animals.

### DXA

DXA scans were acquired at 52-wk (prior to OVX or sham surgery), 56-wk (prior to Ang II or vehicle infusion), and 62-wk (prior to euthanasia) of age to measure whole-body aBMD, fat mass, and lean mass. Anesthetized mice were scanned in the prone position using a Faxitron UltraFocus (Hologic) after the instrument was calibrated as previously described.^32^ ROI excluded the osmotic minipump and head.

### Blood pressure

Blood pressure was measured using a non-invasive tail-cuff system (MC4000 Blood Pressure Analysis System, Model MC4000MSP, Hatteras Instruments). Measurements were done prior to DXA scanning in which the mice were acclimatized to the tail-cuff procedure for 2 consecutive days before BP measurement. All measurements were conducted at the same time of day (9 am to 12 pm) over a 3-d period. The mice acclimated to the machine for 5 min before any measurements to reduce stress and increase body temperature for more accurate results. A minimum of 20 BP readings were averaged from each animal at each time point to obtain final readings of systolic BP and heart rate.

### Micro-CT (μCT)

All μCT analyses were performed on a Scanco μCT50 system (Scanco Medical AG). Femurs (mid-diaphysis and distal metaphysis) and sixth lumbar (L6) vertebral bodies (VBs) were secured in a μCT specimen tube holder containing phosphate buffered saline (PBS). The femurs were scanned at an isotropic voxel size of 6 μm (70 kVp, 114 μA, 8 W; 0.1 mm Al filter, 1024 samples per 1000 projections per 360° rotation; and 300 ms integration time at 2 frame averages). The L6 VB were scanned at an isotropic voxel size of 12 μm (55 kVp, 200 μA; 11 W; 0.5 mm Al filter; BH: 1200 mgHA/cm^3^; 852 samples per 1000 projections per 360° rotation; and 600 ms integration time at 2 frame averages). A beam hardening correction factor (BH: 1200 mg HA) was used during image acquisition to reduce any signal artifacts per manufacturer recommendations. Upon applying a Gaussian noise filter to the image stack (sigma = 0.2 and support = 1), we segmented the bone from soft tissue and air using the global threshold for cortical bone (between 900.5 and 2229.3 mgHA/cm^3^) and trabecular bone (between 467.7 and 2229.3 mgHA/cm^3^). The scans were evaluated as previously described after reconstructing the raw image stack.^33^^,^^34^

### Micro-finite element analysis (μFEA)

All vertebral body (VB) scans were modeled and assessed using the Scanco FE-software (fe_solve3, v1.13, Scanco Medical AG). The ROI was defined by the 3-dimensional reconstruction of the L6 VB excluding the end plates and transverse processes as previously described.^35^ The estimated failure force in compression was the reaction force that caused 2% of the bone volume to exceed 0.7% equivalent strain as previously described.^34^^,^^35^

### Mechanical testing

All mechanical testing was done using a material testing system (Dynamight 8841, Instron). Intact left femurs were placed in a 3-point bending configuration such that the anterior side faced down and medial side faced forward. The span between the lower support was 8 mm. Bones were hydrated before loading at a rate of 3.0 mm/min until failure. A previously described MATLAB script was used to analyze the data.^33^

The contralateral right femurs were micro-notched on their posterior side.^33^ Prior to testing, the notch was scanned by a Scanco μCT 50 system (Scanco Medical) with an isotropic voxel size of 6 μm. These specimens were subjected to 3-point bending at a loading rate of 0.5 mm/min and with a varying span equal to 4 times the anterior–posterior width. The crack initiation toughness was determined from the maximum load and the notch geometry as described previously.^36^^,^^37^

L6 VBs were loaded at 3 mm/min in compression. The end plates were removed, and transverse processes were trimmed. A 2.0 mm diameter, flat, cylindrical platen was used to compress the VB on another platen with a moment relief. Vertebral body strength was the ultimate load that the bone experienced.

### ^1^H-nuclear magnetic resonance relaxometry

Prior to micro-notching, we measured bound water concentration in each right femur as done previously.^33^ After weighing each femur in the air (wet mass), the bone was weighed while submerged in water to estimate the volume of the femur. Samples were allowed to air-dry for another minute before being sealed in a glass NMR tube (Part no. 662001075, Wilmad Labglass) and inserted into our custom low-proton, loop-gap style radiofrequency (RF) coil. A microsphere containing 21.2 μL of water was placed next to the bone inside the coil. Using a 4.7-T horizontal bore magnet (Varian Medical Systems), 10 000 echoes at 100 μs echo spacing were acquired for 90° RF pulses with a duration of 6 μs or 180° at 12 μs. The data was processed and analyzed as previously reported.^33^

### Serum analysis

1 mL syringes were heparinized before collecting blood from the heart’s left ventricle. Samples clotted for 30 min at room temperature before centrifugation. The serum was aliquoted for quantifying circulating bone remodeling serum markers via ELISA as described by the manufacturer’s instructions (Immunodiagnostics): C-terminal telopeptide 1 (CTX-1), tartrate-resistant acid phosphatase 5b (TRAcP 5b), and procollagen type 1 N-terminal propeptide (P1NP).

### Histology and static histomorphometry

The third and fourth lumbar VB samples were fixed in 10% neutral buffered formalin at room temperature for 48 h. Samples were stored at 4 °C in 70% EtOH before decalcification in 10% EDTA (pH 7.4) until completely decalcified as verified using a KUBET X-ray scanner. The samples were cleared and embedded in paraffin before being further processed at the Washington University St. Louis Histology Core to be sectioned and stained. Five-micrometer transverse sections were subjected to H&E staining or tartrate-resistant acid phosphatase (TRAP) staining. Osteoclasts and osteoblasts were quantified using the Bioquant Analysis System, following standard nomenclature from the American Society of Bone and Mineral Research.^38^

### Statistical analysis

Basing the sample size on preliminary differences in ultimate stress (effect size *f* = 0.789) and trabecular BV/TV (effect size *f* = 0.645) between vehicle-infused mice and Ang II-infused mice, 12 mice per group is sufficient to reject the null hypothesis that bone strength and BV/TV do not depend on hypertension, ovariectomy, and their interaction (G*Power 3.1 calculator for numerator degrees of freedom = 3). The probability of type I error (α) and probability of type II error (1-β) in this power calculation were 0.05 and 0.95, respectively. To assess whether longitudinal changes in BP, body mass, aBMD, and fat mass depended on group, we used a mixed-effects model with time, surgery, infusion, and their interactions as fixed effects and with mouse as a random effect. For measurements obtained at the end of the study or after euthanasia, two-way analysis of variance (ANOVA) was used to determine if surgery, infusion, and/or their interaction significantly affected a property (Prism v10.4, GraphPad Software, LLC). In the event the residuals did not pass either the Anderson–Darling test for normality or the Spearman’s test for heteroscedasticity, we used the Aligned Rank Transform ANOVA in R (R version 4.4.3). In post hoc analysis (Prism v10.4, GraphPad Software, LLC), the Holm-Šídák’s test or the non-parametric Dunn’s test determined if pairwise comparisons between groups were significant. In either case, the *p*-value was adjusted assuming 1-3 families: Vehicle (Veh) vs Ang II-hypertension (HTN) within Sham and within OVX (when hypertension was significant); Sham vs OVX within Vehicle and within HTN (when surgery was significant); and Sham-Vehicle vs OVX-HTN and Sham-HTN vs OVX-Vehicle (when the interaction was significant). The non-parametric comparison test occurred when the data of a given group did not pass the Anderson–Darling test (normality assumption) or did not pass Bartlett’s test for differences in standard deviation between groups (homogeneous of the variance assumption).

## Results

### Angiotensin II caused hypertension in OVX mice, but it did not worsen the OVX-related decline in whole-body bone mass

**Figure 2:**
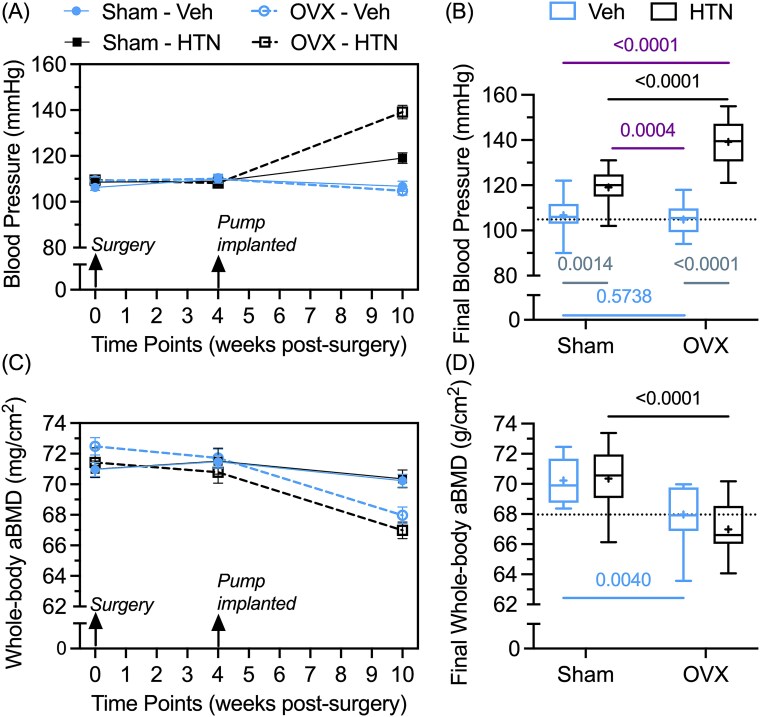
Longitudinal measurements of blood pressure (BP) and areal BMD at 3 time points and final measurements at euthanasia for each of the 4 groups. Blood pressure increased following the infusion of angiotensin II to induce hypertension (HTN) (A) such that BP was highest in OVX mice (B). Whole-body areal BMD (aBMD) declined in only the OVX mice (C) with no difference in aBMD between the Vehicle and HTN groups (D). ANOVA *p*-values reported in Table 1 for (A) and (C) or Table 2 for (B) and (D).

Time point (baseline being day of surgery, pump implantation being 4 wk later, and euthanasia 10 wk later), infusion (Vehicle or HTN), and surgery (Sham or OVX) as well as their interactions significantly affected BP (Table 1, Figure 2A and B), but only time point of the measurement, and the interaction between surgery and time point significantly affected whole-body aBMD (Table 1, Figure 2C and D). There was no significant difference in the BP between Sham and OVX, which remained normal in vehicle-infused mice; while OVX-HTN mice had significantly higher BP compared to Sham-HTN mice (Figure 2B). OVX, not HTN, significantly lowered whole-body aBMD, irrespective of the infusion group (Figure 2D).

**Table 1:** *p*-values from analysis of variance of longitudinal measures over time periods: surgery at 52-wk of age, pump implanted at 56-wk of age, and pre-euthanasia at 62-wk of age. Body mass was measured weekly between 52-wk and 62-wk of age.

**Mixed effects model** ^**a**^	**Body mass**	**Systolic BP**	**Whole-body aBMD**	**Fat mass**	**Lean mass**
Surgery	0.1991	0.0017	0.1002	0.8904	0.1826
Infusion	0.0570	<0.0001	0.3168	0.5991	0.5324
Time point	<0.0001	<0.0001	<0.0001	<0.0001	0.0002
Surgery × infusion	0.8508	0.0043	0.2271	0.9455	0.8869
Surgery × time point	0.0317	0.0016	<0.0001	0.1282	0.0112
Infusion × time point	0.1347	<0.0001	0.9849	0.0289	0.7823
Surgery × infusion × time point	0.4783	<0.0001	0.9982	0.9179	0.7788

a Fixed effects were Surgery (Sham or OVX) and Infusion (Vehicle or Ang II-hypertension). There were missing measurements over the Time points (0, 4, or 10 wk post-surgery per Figure 1), so mouse was modeled as a random factor.

**Figure 3:**
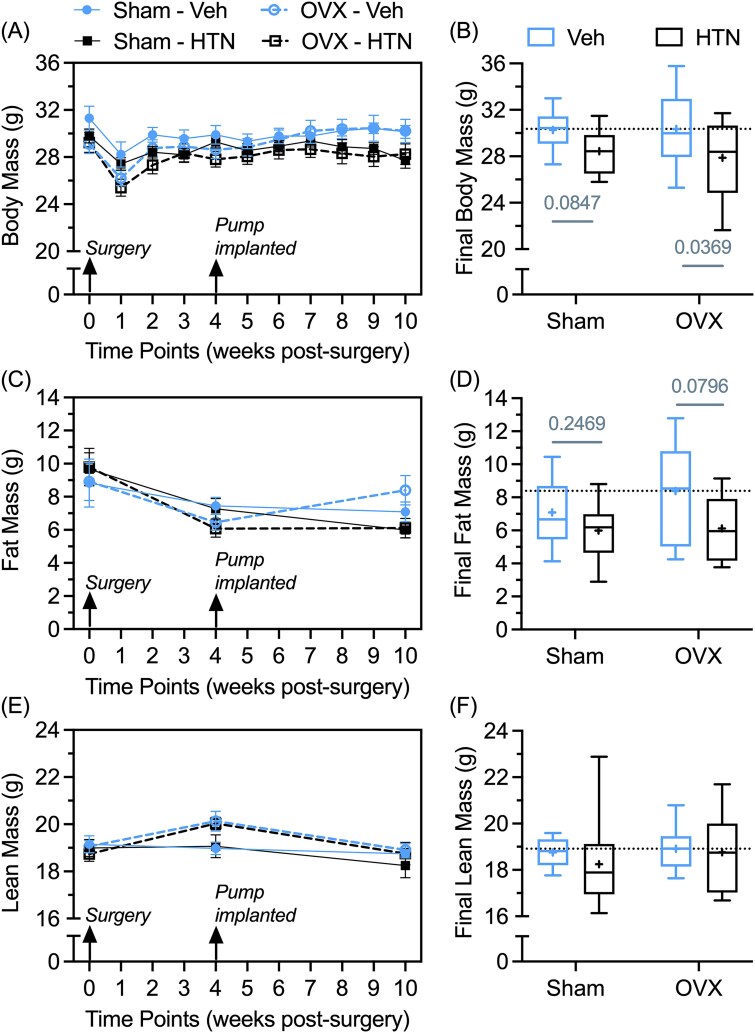
Longitudinal measurements of body mass, fat mass, and lean mass at 3 time points and final measurements at euthanasia for each of the 4 groups. After decreasing post-surgery, body mass stabilized over the 10-wk study period (A) such that mice in the HTN group weight less than mice in the vehicle group at end-point (B). Fat mass by DXA initially decreased in all 4 groups after surgery, but it varied among the 4 groups during the infusion period (C). HTN, not OVX, affected fat mass, which was non-significantly lower in HTN than in Vehicle group (D). Lean mass increased 4 wk after OVX, but it returned to the levels of the Sham groups (E). At end-point, there was no significant difference in lean mass among the 4 groups (F). ANOVA *p*-values reported in Table 1 for (A), (C), and (E) or Table 2 for (B), (D), and (F).

After 1-wk post-surgery (Sham or OVX), mice significantly gained weight over time (Figure 3A); however, there was a significant interaction between surgery and time point (Table 1). When averaged over the final 3 wk of life, the body weight was significantly affected by hypertension (Table 2). Mice in the OVX-HTN group had significantly lower body mass than mice in the OVX-Vehicle group (Figure 3B). The time point related to the advancing age of the animal at surgery, at pump implantation, and at euthanasia and its interaction with infusion group significantly affected DXA-derived fat mass (Table 1, Figure 3C); however, the lower final fat mass between Vehicle and HTN within each surgical group was not strictly significant in the post hoc pairwise comparisons (Figure 3D). The time point and its interaction with surgery significantly affected DXA-derived lean mass (Table 1, Figure 3E), but there was no effect on the final lean mass by post hoc analysis (Table 2, Figure 3F).

OVX affected the post-mortem uterus mass and spleen mass, while HTN significantly affected heart mass (Table 2). As expected, the uterus mass was significantly lower in OVX than in Sham within each infusion group, while the heart mass was significantly higher in HTN than in Vehicle within each surgical group (Table 3). The higher spleen mass with OVX surgery was not significantly different in post hoc pairwise comparisons within control and HTN groups (Table 3). Moreover, there were no differences in femur length or anterior-posterior width (Table 2).

### Hypertension and ovariectomy independently decreased bone formation, while just hypertension increased bone resorption

Hypertension affected both CTX-1, a serum marker of bone resorption, and TRAcP 5b, a serum marker of osteoclast activity (Table 2). The marker of resorption and osteoclast activity was higher in HTN than in Vehicle, irrespective of surgery group (Table 3). OVX and HTN also affected P1NP, a serum marker of bone formation. There was a significant reduction in P1NP levels in OVX, irrespective of hypertension, as well as in OVX-HTN compared to OVX-Vehicle (Table 3). The interaction term was not significant for any of these measures, but the lowest measurement of P1NP occurred in the estrogen deficiency-hypertension group suggesting HTN and OVX surgery may be additively affecting bone formation.

Neither surgery nor infusion significantly affected number of osteoclasts on the bone surface (N.Oc/BS) within the L3 or L4 VB; while only HTN affected the number of osteoblasts on the bone surface (N.Ob/BS) (Table 2). N.Ob/BS was lower in hypertensive mice than in normotensive mice, but the differences between Vehicle and HTN within surgery group were not strictly significant in the pairwise comparisons (Table 3).

### Hypertension and estrogen deficiency each decreased trabecular thickness and tissue mineral density, while OVX weakened the lumbar vertebra

For the aged female mice in the present study, there was minimal trabecular bone in the distal femur metaphysis (Figure S1A and B). As such, neither OVX, hypertension, nor their interaction affected trabecular bone volume fraction (BV/TV in Table 2). Nonetheless, OVX and its interaction with infusion group significantly affected trabecular thickness (Tb.Th) and trabecular tissue mineral density (Tb.TMD), with no other trabecular parameters being different among the 4 groups (Table 2, Table S1 and Figure S1C). While there was no significant difference in Tb.Th between Sham and OVX within the Vehicle group, Tb.Th was significantly lower in OVX-HTN compared to Sham-Vehicle and to Sham-HTN (Figure S1C). Likewise, the effect of OVX on Tb.TMD depended on infusion group such that Tb.TMD was only lower in OVX compared to Sham when mice had hypertension (Table S1).

**Table 2:** *p*-values from two-way analysis of variance for end-point measurements in which OVX surgery caused estrogen deficiency and Ang II infusion caused hypertension.

**Measurement**	**Surgery**	**Infusion**	**Interaction**
**Whole body**			
**Blood pressure**^**a**^	0.0006	<0.0001	<0.0001
**Areal BMD**	<0.0001	0.4206	0.3021
**Body mass**	0.7374	0.0048	0.6542
**Fat mass**^**a**^	0.3697	0.0291	0.4986
**Lean mass**	0.3866	0.3969	0.6519
**Soft tissues**			
**Uterus mass**^**a**^	<0.0001	0.3811	0.4849
**Heart mass/body mass**^**a**^	0.6619	0.0016	0.9199
**Spleen mass/body mass**^**a**^	0.0248	0.2247	0.6540
**Femur dimensions**			
**Length**	0.1574	0.6501	0.6269
**Anterior posterior width**	0.1339	0.3272	0.9625
**Serum analysis**			
**P1NP**	0.0002	0.0017	0.2617
**CTX-1**^**a**^	0.7808	0.0004	0.7335
**TRAcP 5b**^**a**^	0.0669	<0.0001	0.8851
**Histology**			
**Number of osteoclast by bone surface**^**a**^	0.0509	0.1879	0.5838
**Number of osteoblast by bone surface**^**a**^	0.3934	0.0048	0.9543
**Cortical structure (left femoral mid-diaphysis)**			
**Cortical area**	0.0010	0.0543	0.3478
**Cortical thickness**^**a**^	<0.0001	0.0949	0.2302
**Cortical porosity**^**a**^	0.0184	0.7272	0.0866
**Medullary volume**	<0.0001	0.9029	0.1637
**Minimum moment of inertia**	0.9460	0.0460	0.9281
**Section modulus**	0.1485	0.0406	0.5223
**Polar moment of inertia**	0.3279	0.1039	0.9482
**Total area**	0.0753	0.2072	0.4213
**Cortical tissue mineral density**	<0.0001	0.6056	0.0017
**Cortical volumetric BMD**	<0.0001	0.4110	0.0013
**Biomechanics (femur mid-diaphysis)**			
**Ultimate force**	0.0259	0.4504	0.0151
**Yield force**^**a**^	0.0015	0.1411	0.0809
**Work-to-failure**^**a**^	0.0429	0.4480	0.0362
**Post-yield displacement**^**a**^	0.1410	0.2583	0.5898
**Matrix analysis of whole bone**			
**Bound water volume fraction**	<0.0001	0.4340	0.3475
**Fracture toughness testing (mid-diaphysis)**			
**Fracture toughness**	0.9765	0.7921	0.8000
**Trabecular properties (distal femur metaphysis)**			
**Bone volume fraction**^**a**^	0.3314	0.6566	0.6449
**Trabecular number**	0.4518	0.8289	0.0801
**Trabecular thickness**	0.0007	0.8913	0.0494
**Trabecular separation**	0.3688	0.8883	0.0944
**Connectivity density**^**a**^	0.3268	0.0852	0.2716
**Structure model index**	0.6965	0.0622	0.3154
**Trabecular tissue mineral density**	<0.0001	0.1452	0.0032
**Trabecular microarchitecture (sixth lumbar vertebral body)**			
**Bone volume fraction**	0.0017	0.0835	0.6858
**Trabecular number**	0.1449	0.2048	0.9858
**Trabecular thickness**	<0.0001	0.0036	0.3105
**Trabecular separation**	0.1609	0.1043	0.9555
**Connectivity density**^**a**^	0.3096	0.1197	0.5964
**Structural model index**^**a**^	0.0034	0.6016	0.8085
**Trabecular tissue mineral density**	0.0011	0.0002	0.9984
**Bone area**	0.0239	0.2021	0.4914
**Micro-finite element analysis (sixth lumbar vertebral body)**			
**Estimated failure load**	0.0006	0.0051	0.8535
**Biomechanics (sixth lumbar vertebral body)**			
**Ultimate force**	0.0049	0.1953	0.9668
**Yield force**	0.0500	0.3493	0.8334

a Parameters violated normality or homoscedasticity, so they were analyzed using non-parametric ANOVA.

**Figure 4:**
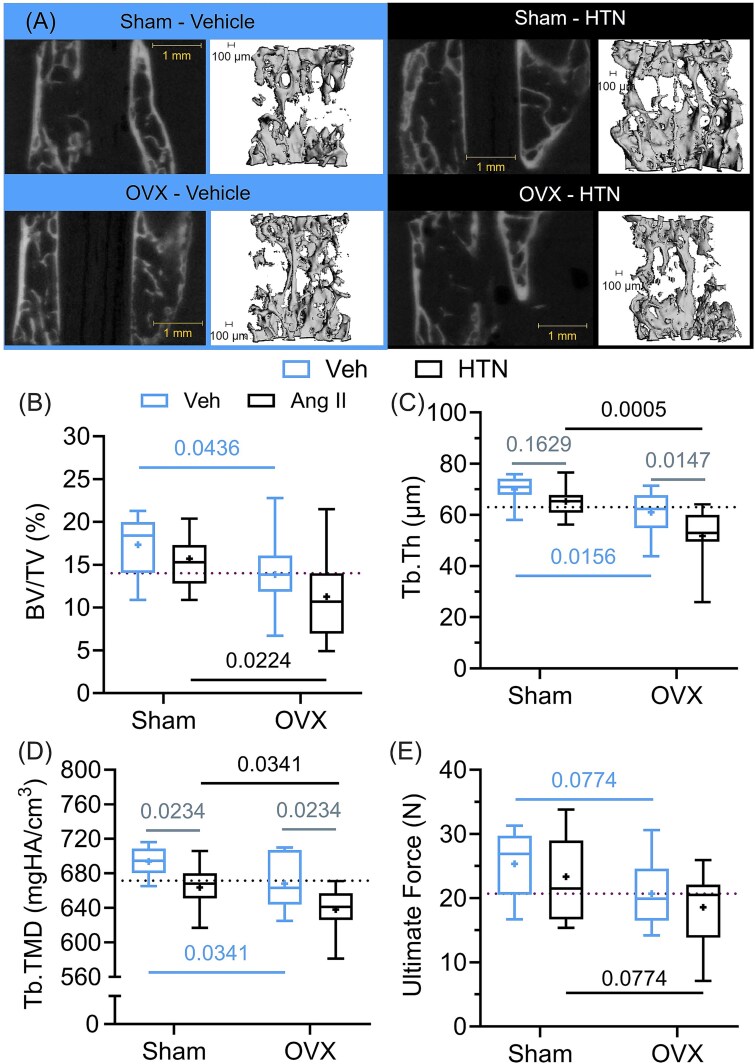
Effect of ovariectomy and hypertension on trabecular architecture and strength of the sixth lumbar vertebral body (L6 VB). The L6 VB is a trabecular-rich site such that the amount of trabecular and its architecture influence its strength (A). OVX, but not HTN, decreased trabecular bone volume fraction (B), while both OVX and HTN decreased trabecular thickness (C). Likewise, they both independently decreased trabecular tissue mineral density (D). While the post hoc, pairwise comparisons were not strictly significant for the effect of OVX on the compressive strength of the L6 VB, the OVX-HTN group had the lowest ultimate force (E). ANOVA *p*-values reported in Table 2 for all parameters.

The aged female mice had more trabecular bone (BV/TV between 13.2% and 16.1%; Figure 4A and Table S1) in the L6 VB than in the distal femur metaphysis, and both HTN and OVX each significantly affected Tb.Th, and Tb.TMD with no significant interactions (Table 2). OVX only significantly affected BV/TV and bone area (Table 2). In the pairwise comparisons, BV/TV, Tb.Th, and Tb.TMD were significantly lower in OVX than in Sham within Vehicle and within HTN (Figure 4). Bone area was not different by

pairwise comparison (Table S1). No other μCT-derived parameters were affected, except the estimated failure load by μFEA was lower in OVX than in Sham, irrespective of infusion group (Table S1). Even though the effect of OVX on trabecular architecture did not depend on HTN for the L6 vertebra, mice in the OVX-HTN had the lowest median and mean values of trabecular bone architecture relative to the other 3 groups (Figure 4B and C). Estrogen deficiency, but not HTN, also affected the ultimate force that the L6 VB endured during compression testing (Table 2). This measure of compressive strength was lower in OVX than in Sham within each infusion group, but the adjusted *p*-value was not strictly significant (Figure 4E). Nonetheless, the combination of OVX and HTN resulted in the lowest mean of compressive strength of L6 VB (ie, ultimate force; Figure 4E).

**Table 3:** Soft tissue parameters, femur dimensions, serum markers of bone turnover, and static histology quantification among the 4 groups with adjusted *p*-values from Holm-Šídák’s pairwise comparisons.

**Surgery group**		**Sham**				**Veh vs HTN**	**OVX**				**Veh vs HTN**	**Sham vs OVX**	
**Infusion group**		**Vehicle**		**HTN**			**Vehicle**		**HTN**			**Veh**	**HTN**
**Property**	Units	Mean	SD	Mean	SD	*p*-value	Mean	SD	Mean	SD	*p*-value	*p*-value	*p*-value
**Organ mass**		*n* = 12		*n* = 11			*n* = 12		*n* = 13				
**Uterus mass**	g	0.217	0.063	0.212	0.084	NA^a^	0.046	0.032	0.037	0.018	NA	.0001^b^	<.0001
**Heart mass** ^**c**^	mg/g	0.478	0.068	0.549	0.084	.0445	0.479	0.0678	0.570	0.101	.0156	NA	NA
**Spleen mass** ^**c**^	mg/g	0.425	0.161	0.436	0.102	NA	0.507	0.192	0.555	0.161	NA	.4869^b^	.1433
**Femur dimensions**		*n* = 11		*n* = 11			*n* = 12		*n* = 13				
**Length**	mm	15.10	0.32	15.2	0.27	NA	15.30	0.38	15.30	0.39	NA	NA	NA
**Anterior-posterior width**	mm	1.43	0.04	1.41	0.05	NA	1.45	0.07	1.44	0.05	NA	NA	NA
**Serum markers**		*n* = 12		*n* = 11			*n* = 12		*n* = 12				
**P1NP**	ng/mL	199.4	52.2	174.4	26.6	.1069	165.5	38.7	115.0	30.5	.0151	.0083	.0006
**CTX-1**	ng/mL	17.9	4.6	27.0	8.4	.0142	18.0	5.9	26.6	10.6	.0436^b^	NA	NA
**TRAcP 5b**	U/L	2.50	1.03	3.96	1.34	.0167^b^	1.75	0.54	3.50	1.15	.0004	NA	NA
**Histology of L3-L4 VB**		*n* = 12		*n* = 9			*n* = 11		*n* = 12				
**N.Oc/BS**	#/mm	0.23	0.20	0.34	0.25	NA	0.38	0.27	0.39	0.19	NA	NA	NA
**N.Ob/BS**	#/mm	1.01	0.44	0.55	0.37	.0838	1.13	0.54	0.78	0.59	.1815^b^	NA	NA

a Not applicable (NA) because the fixed effects in the ANOVA was not significant (Table 2).

b Adjusted *p*-values come from Dunn’s pairwise comparison because group values violated normality assumption or homoscedastic assumption.

c Mass of tissue per mass of mouse.

### Hypertension enhanced the negative effect of ovariectomy on cortical structure, matrix-bound water, and bending strength of the femur mid-diaphysis

**Figure 5:**
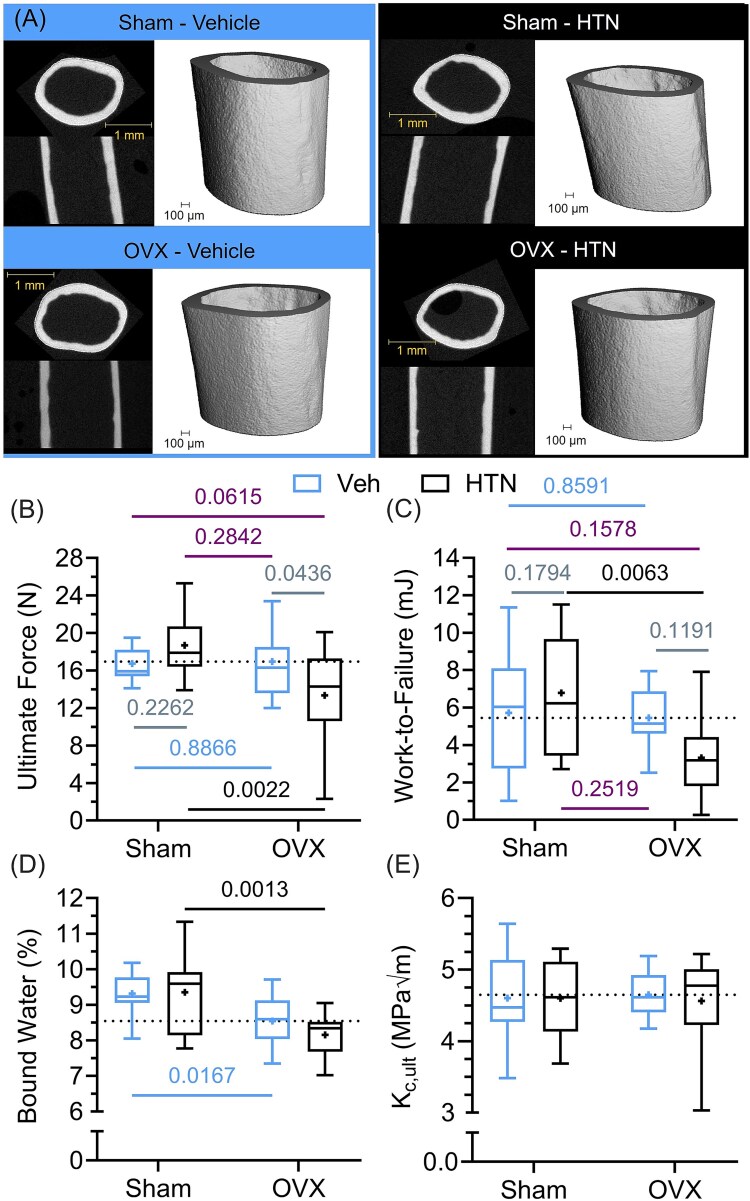
Effect of ovariectomy and hypertension on the fracture resistance and bound water of the femur. As determinants of bone strength, cortical area and cortical thickness were determined from μCT images of the femur mid-diaphysis (A). HTN reduced the ultimate force (bending strength) of the femur mid-diaphysis only in OVX mice (B). This was also the case for work-to-failure of the femur (C). Only OVX decreased bound water per bone volume (D), while neither OVX nor HTN affect crack initiation toughness (E). ANOVA *p*-values are reported in Table 2 for all parameters.

As for cortical bone of the femur mid-diaphysis (Figure 5A), OVX only affected the cross-sectional bone area (Ct.Ar), cortical thickness (Ct.Th), cortical porosity (Ct.PO), and medullary volume (Ma.V) while hypertension alone affected the cross-sectional distribution of bone tissue relative to the minor (ie, anterior-posterior) axis (*I*_min_) and the related section modulus (Table 2). In the pairwise comparisons, Ct.Ar was significantly lower in OVX-HTN mice compared to Sham-HTN mice. The hypertension-related decrease in *I*_min_ and section modulus was not significant by post hoc*,* pairwise comparison (Table 4). OVX decreased Ct.Th and increased Ma.V with the difference between Sham and OVX being more pronounced in HTN than in Vehicle control (Table 4). The effect of OVX on tissue mineral density (TMD) and volumetric BMD (vBMD) of cortical bone (Ct) depended on infusion (significant interaction in Table 2) such that Ct.TMD and Ct.vBMD were significantly lower in OVX-HTN compared to OVX-Vehicle, Sham-HTN, and in Sham-Vehicle (Table 4).

**Table 4:** Micro-CT-derived and biomechanical parameters among the 4 groups with adjusted *p*-values from Holm-Šídák’s pairwise comparisons.

**Surgery group**		**Sham**				**Veh vs** **HTN**	**OVX**				**Veh vs** **HTN**	**Sham vs OVX**		**Sham-Veh vs** **OVX-HTN**
**Infusion group**		Vehicle		HTN			Vehicle		HTN			Veh	HTN	
**Property** ^**a**^	**Units**	Mean	SD	Mean	SD	*p*-value	Mean	SD	Mean	SD	*p*-value	*p*-value	*p*-value	*p*
**μCT**		*n* = 11		*n* = 11			*n* = 11		*n* = 13					
**Ct.Ar**	**mm** ^**2**^	0.785	0.051	0.767	0.060	NA^**b**^	0.739	0.067	0.687	0.063	NA	.0783	.0046	NA
**Tt.Ar**	**mm** ^**2**^	2.10	0.12	2.03	0.13	NA	2.14	0.14	2.12	0.11	NA	NA	NA	NA
***I***_**min**_	**mm** ^**4**^	0.162	0.019	0.150	0.018	.2409	0.162	0.024	0.149	0.021	.2409	NA	NA	NA
**pMOI**	**mm** ^**4**^	0.434	0.051	0.409	0.055	NA	0.420	0.059	0.393	0.047	NA	NA	NA	NA
**Ct.Th**	**mm**	0.169	0.008	0.167	0.012	NA	0.155	0.011	0.145	0.014	NA	.0089	<.0001	NA
***I***_**min**_ **/*c***_**min**_	**mm** ^**3**^	0.219	0.019	0.209	0.020	.3146	0.213	0.023	0.196	0.023	.1439^c^	NA	NA	NA
**Ma.V**	**mm** ^**3**^	2.44	0.15	2.36	0.20	NA	2.59	0.17	2.66	0.16	NA	.0415	.0002	NA
**Ct.Po**	**%**	4.33	0.25	4.21	0.42	NA	4.43	0.33	4.57	0.75	NA	.6311	.0297^c^	NA
**Ct.TMD**	**mgHA/cm** ^**3**^	1321	9	1330	11	.0557	1314	11	1302	10	.0153	.1014	<.0001	<.0001
**Ct.vBMD**	**mgHA/cm** ^**3**^	1264	8	1272	12	.0764	1255	10	1242	12	.0071	.0704	<.0001	<.0001
**Three-point bending**		*n* = 11		*n* = 11			*n* = 11		*n* = 13					
**Yield force**	N	14.3	1.0	15.1	2.6	NA	13.4	3.1	10.8	3.9	NA	.4891	.0015	NA
**PYD**	mm	0.432	0.313	0.353	0.147	NA	0.334	0.087	0.224	0.119	NA	NA	NA	NA

a Properties come from μCT evaluations of the distal femur metaphysis.

b Not applicable (NA) because the fixed effects or interaction in the ANOVA were not significant (Table 2).

c Adjusted *p*-values come from Dunn’s pairwise comparison because group values violated normality assumption or homoscedastic assumption.

The effect of OVX-estrogen deficiency on bending strength depended on infusion group (ie, significant interaction term for ultimate force in Table 2). The ultimate force that the mid-diaphysis endured during 3-point bending was significantly lower in OVX-HTN than in OVX-Vehicle and in Sham-HTN mice (Figure 5B). In other words, hypertension reduced cortical bone strength in OVX mice and OVX reduced cortical bone strength in hypertensive mice. OVX alone reduced yield force of the mid-diaphysis, thereby reducing cortical bone strength during HTN (Table 4). Neither OVX nor hypertension significantly affected post-yield displacement, a measure of brittleness. OVX lowered work-to-fracture, but only in the hypertensive mice (Figure 5C). Within the bone tissue, OVX decreases the level of bound water with greatest difference occurring in the hypertensive mice (Figure 5D). This effect did not translate to an OVX-related reduction in the ability of the cortical bone to resist crack growth (*K*_c,ult_; Figure 5E).

## Discussion

Osteoporosis remains underdiagnosed and undertreated for a variety of reasons^39^^,^^40^ including the lack of symptoms during bone loss until a fracture occurs. There are also challenges in the treatment of hypertension related to insufficient detection of the disease and sub-optimal BP control as the disease progresses.^41^ This is particularly true for postmenopausal women who are at risk of a fragility fracture^2^ and developing hypertension^4^^,^^5^ compared to age-matched men. Therefore, we induced hypertension 4 wk after ovariectomy in middle-aged mice to determine if these 2 conditions additively affect bone mass and strength. In doing so, we found that the induction of “menopause” by ovariectomy weakened cortical bone when middle-aged mice were hypertensive. OVX and HTN independently affected trabecular thickness and TMD, and the combination resulted in the lowest mean value of compressive strength. Such findings support combining surgically induced estrogen deficiency and continuous angiotensin II infusion to investigate strategies that could effectively treat 2 co-occurring chronic diseases, hypertension and osteoporosis, that increase fracture risk.

Performing OVX in older mice after reproductive senescence^27^^,^^28^ mimics menopausal age in women. However, OVX-induced loss in trabecular bone fraction (BV/TV) diminishes as the age of the mouse at the time of surgery increases.^42^ As such, OVX alone at 12 mo of age did not significantly reduce BV/TV and strength of the L6 VB (Figure 4) nor reduce BV/TV of the distal femur metaphysis (Table S1, Figure S1) as might be expected for younger mice subjected to OVX.^43^^,^^44^ The effect of hypertension on these parameters is unknown for young female mice, but we found that hypertension weakens the L6 VB in young (ie, HTN between 13 and 19 wk), male mice.^26^ Although not as pronounced as this previous study using male C57BL/6J mice, the combination of OVX and Ang II weakened the L6 VB of 12-mo-old, C57BL/6J mice in compression (Figure 4E). This was indicated by the μFEA (Table S1) that accounts for thinner, less dense trabeculae causing higher tissue-level strains, which in turn favors failure of the bone. The ultimate load from the mechanical, load-to-failure tests of VBs did not depend on the interaction between surgery and infusion (Table 2), but this measurement of strength was lowest in the OVX-HTN group. The percent drop in ultimate force between Sham-Vehicle mice and OVX-HTN mice was 26.8%, compared to a percent drop of 18.5% between Sham-Vehicle and OVX-Vehicle.

With respect to cortical bone, ovariectomy in 4-mo-old mice was found to reduce cortical thickness and cortical TMD^17^ as well as to moderately lower bending strength of the mid-diaphysis.^45^ The effect of OVX on bending strength in aged mice is unknown. Likewise, the effect of hypertension on bending strength in female mice of any age is unknown to the best of our knowledge. We found that the effect of HTN on bending strength (ie, ultimate force during 3-point bending) depended on ovariectomy (significant interaction for ultimate force in Table 2) such that hypertension only reduced strength of cortical bone in OVX mice or ovariectomy only reduced this strength in hypertensive mice (Figure 5B). This dependency suggests the differential effects of OVX (low cortical thickness) and HTN (low moment of inertia) on bone structure are required to confer weakness to the cortical bone of the femur mid-diaphysis, but it may also require the reduction in cortical volumetric BMD that only happened when both OVX and HTN occurred.

Estrogen-deficiency is known to increase the production of pro-inflammatory and pro-osteoclastic cytokines like TNF alpha (TNFα), RANKL, interleukin (IL)-6, macrophage-colony stimulating factor (M-CSF/CSF-1), among others.^46^ Not only has osteoclastogenesis been observed in the context of hypertension, but these cytokines also play a significant role in the progression of the disease.^26^^,^^47^ Previously, we found that blocking M-CSF receptor signaling blunted HTN-induced bone loss by preventing expansion of monocytes and macrophages.^26^ Selective deletion of the soluble M-CSF isoform or neutralization of M-CSF attenuates OVX-induced bone loss.^48^^,^^49^ These observations suggest that M-CSF signaling may be a common mechanism by which hypertension and loss of estrogen drive osteoclastogenesis and bone loss. Additionally, T cells contribute to osteoclastogenesis,^46^ ovariectomy-induced bone loss,^50^^,^^51^ and the genesis of hypertension.^18^ Specifically, IL-17A production by T cells contributes to both hypertension and osteoporosis.^18^^,^^26^^,^^51^ Thus, inhibiting M-CSF (CSF-1) and/or IL-17A may be a potential target for treating both diseases. The present study supports the combination of OVX and hypertension in middle aged mice as a pre-clinical model to investigate mechanisms of inflammatory pathways that cause a loss in bone strength, since it caused the greatest loss in whole-bone strength at both a cortical and a trabecular-rich site.

Studies in young female mice report increased osteoblastogenesis and bone formation rate with OVX, suggesting that bone resorption exceeds bone formation during estrogen loss.^52–54^ In the present study, hypertension, not OVX, increased markers of bone resorption and osteoclast activity (CTX-1 and TRAcP 5b; Table 2). This did not translate to lower osteoclast number in the VB in estrogen-deficient, hypertensive mice (Table 2) as might be expected from OVX studies in young female mice and hypertension studies in young male mice. OVX decreased serum P1NP levels with the most pronounced decrease occurring with hypertension (Table 3). Serum markers are systemic and a product of cell number and activity, so potentially only activity is affected by estrogen-deficiency and hypertension in middle-aged mice. Alternatively, we only analyzed a lumbar VB per mouse, and possibly older animals require more tissue sections to observe differences than is required for younger animals, leading to the discordance between serum markers of bone turnover and numbers of bone cells on the bone surface.

Ang II-hypertension can decrease osteoblast number in the vertebra of young male mice^26^ like seen in this study (Table 2). Some studies suggest that direct signaling via the Ang type I or II receptors (AT1R or AT2R) on osteoblasts affects differentiation and activation such that inhibition of these receptors increased pro-osteoblastic gene expression and calcium deposition in culture, decreased production of pro-osteoclastic cytokines in culture, and improved bone parameters in vivo*.*^25^^,^^55^ In vitro studies implicate that Ang II directly stimulates osteoclast formation from macrophages^56^ and/or promotes RANKL production by osteoblasts,^55^ though these studies treated cells with supraphysiological concentrations of Ang II relative to circulating levels.^57^ While we cannot rule out a direct effect of Ang II on osteoblasts or osteoclasts, we observed bone loss in the DOCA-salt model in young male mice, a model of low circulating renin and Ang II.^58^ To further elucidate the role of hypertension on bone loss, alternative models of hypertension could be used like the DOCA-salt model, the Goldblatt model, or the *N*^ω^-nitro-l-arginine (L-NAME)-high salt model (Table 5).

**Table 5:** Common murine models of hypertension.

**Model of hypertension**	**Manipulation**	**Appropriate control**	**Typical model length of time**	**Can it be elongated**	**Mechanism of action**	**Bone studies**
**Ang II** ^**a**^ **infusion**	Subcutaneous implantation of osmotic minipump	Subcutaneous osmotic minipump that infused diluent of Ang II	2 wk or 4 wk of infusion	6 and 8 wk have been used	Increased circulating Ang II	^26^
**Deoxycorticosterone DOCA** ^**b**^ **acetate-salt**	Uninephrectomy, subcutaneous DOCA pellet, 1% NaCl in the drinking water	Uninephrectomy and subcutaneous implantation of control pellet	3 wk (using 21-d release pellet)	60- or 90-d release pellets available	Increased plasma volume	^26^
**Goldblatt model (1 kidney 1 clip)**	Uninephrectomy and clip placed at the remaining renal artery	Uninephrectomy	2-3 wk	No reports of longer, but probably can be elongated unless humane end-points are met	Renal ischemia increases plasma volume	None
**Goldblatt model (2 kidney 1 clip at the renal artery)**	Clip placed at the renal artery	Open and expose descending aorta and then close	2-3 wk	No reports of longer, but probably can be elongated unless humane end-points are met	Ischemic kidney produces increased renin	None
**Goldblatt model (2 kidney 1 clip at the abdominal aorta)**	Clip placed on the descending aorta between the left and right renal arteries	Open and expose descending aorta and then close	2-3 wk	No reports of longer, but probably can be elongated unless humane end-points are met	Ischemic kidney produces increased renin	None
**L-NAME** ^c^ **-high salt**	2 wk of L-NAME 2 wk of normal diet 2 wk of 4% NaCl diet	No diet manipulation	2 wk of hypertension	No reports of longer. Telemetry studies would need to be done to monitor continued high BP	Salt sensitive	^59^

a Angiotensin II (Ang II).

b Deoxycorticosterone (DOCA).

c *N*  ^ω^-nitro-l-arginine (L-NAME).

Bone is a hierarchical tissue in which fracture resistance depends on trabecular architecture and cortical structure as well as the relative composition of the extracellular matrix and the arrangement of inorganic mineral within an organic matrix of type 1 collagen, various non-collagen proteins, and water. Genetic mutations, disease, lifestyle, nutrition, and age can negatively affect the composition or arrangement of the bone matrix.^60^^,^^61^ In this study, ovariectomy significantly reduced bound water like seen by Metzger et al. in OVX-rats.^53^ Hypertension did not influence bound water, regardless of ovariectomy. Similarly, hypertension also did not affect bound water in young male C57BL/6J mice.^26^ Additional work is required to understand if the decline in estrogen directly affects matrix-bound water, as suggested by others,^62^ or affects the matrix in such a way that decreases hydrogen bonding between the organic matrix and water (eg, decreases glycosaminoglycans (GAGs) or proteoglycans that bind GAGs). Nevertheless, understanding how estrogen deficiency affects bone matrix quality in pre-clinical models helps to identify and establish new matrix-sensitive techniques for fracture risk assessment.

This study focused on the effects of hypertension and ovariectomy on one, inbred strain of mice. Though limiting genetic variation can be a useful tool for isolating essential mechanisms in disease progression, doing so may mask an important determinant of disease risk. In both fractures and hypertension, genetic variation contributes significantly to risk of occurrence, disease development, and treatment.^17^^,^^63^ Using outbred rodent strains may be more representative of the synergistic or additive effect of the 2 concurrent diseases on the general human population, but would require larger samples sizes and more extensive breeding strategies.^64^ In inbred mouse-strains, ovariectomy is known to have strain-specific effects on cortical and trabecular bone,^65^^,^^66^ suggesting genetic regulation of bone remodeling. Bouxsein et al. suggested that strains with higher bone masses at baseline like BALB/c mice experience larger differences in bone loss after OVX because they have more bone to lose.^67–69^ In turn, repeating this study in 12-mo-old BALB/c mice may lead to greater differences between the 4 groups. Hypertension is also known to have variable pathology based on strain^65^^,^^66^ such that C57BL/6J mice are commonly used to study renin-angiotensin-aldosterone-system-dependent hypertension.^66^ Using a different strain of mice would likely change the pathogenesis of hypertension, potentially changing the relationship between OVX and high BP.

This study lacks a therapeutic intervention for mechanistic insight into the relationship between estrogen-deficiency and high BP, but it does enable such endeavors by demonstrating how the combination of estrogen deficiency and hypertension affects bone. Different signaling pathways can be targeted pharmacologically (eg, antiresorptive, anabolic therapies, antihypertensives, immune modulators, and hormone replacement) in OVX-HTN mice to increase bone strength. Clinical studies suggest that use of antihypertensives like ARBs, selective β-adrenergic receptor blockers, and thiazide diuretics are protective against osteoporosis or fractures.^70^ This suggests that lowering BP protects against bone fragility, but these treatments may not be sufficient to prevent osteoporosis as BP control declines over time.^71^ Additionally, preclinical studies suggest that hormone replacement therapy (HRT) may mitigate the negative effects of OVX^46^ or Ang II-induced hypertension^22^^,^^23^ on bone. Determining whether HRT and/or antihypertensive medications like ACE inhibitors reverse or ameliorate the present effects of OVX and HTN could help to validate the present combination model of bone fragility.

The present study also did not investigate the effect of timing of hypertension relative to the OVX surgery that causes estrogen loss. We initiated hypertension after 4 wk of estrogen deficiency such that bone loss likely had occurred but had not plateaued.^63^ Initiating hypertension closer to or further from the time of OVX could potentially cause more or less bone loss than observed herein. Varying the onset of hypertension after ovariectomy could provide insight into the extent to which hypertension further increases bone fragility (ie, increases fracture risk). Conversely, the onset of hypertension prior to estrogen loss is uncommon and often due to a secondary etiology that is not fully captured by the Ang II model.^30^ By working toward an understanding of how hypertension and estrogen-deficiency act on similar or different molecular pathways, an optimal treatment strategy could potentially be developed to best prevent fractures in this population.

### Conclusion

In the present study, we found that hypertension and ovariectomy have differential effects on cortical and trabecular bone of middle-aged mice. The decrease in the bending strength of femur mid-diaphysis required both OVX-estrogen deficiency and Ang II-hypertension, whereas the decrease in the compressive strength of the sixth lumbar vertebra required OVX with greatest decline occurring with OVX and HTN. Neither experimental manipulation affected the ability of cortical bone to sustain post-yield (ie, inelastic deformation) and to resist crack growth. These findings then suggest that hypertensive, postmenopausal women may be at a higher risk for fractures because of structural- and architectural-dependent weakening of bone. The present pre-clinical model may be useful to determine the mechanisms by which loss of estrogen and hypertension work together to decrease the fracture resistance of bone.

## Supplementary Material

Aged_OVX_HTN_Supplemental_Info_rev_1_v1_ziag101

## Data Availability

Data herein is available upon request.
