# Supplementary material for: Hypertension worsens bone loss and weakens bone in aged ovariectomized mice
Source: JBMR Plus. 2026 Jun 15;10(8):ziag101. doi: 10.1093/jbmrpl/ziag101 (PMC13349054; doi:10.1093/jbmrpl/ziag101)
Supplement: Aged_OVX_HTN_Supplemental_Info_rev_1_v1_ziag101 [file aged_ovx_htn_supplemental_info_rev_1_v1_ziag101.docx]

Table S1: μCT-derived parameters among the 4 groups with adjusted p-values from Holm-Šídák's pairwise comparisons.

| **Surgery group:** | | **Sham** | | | | **Veh vs.** | **OVX** | | | | **Veh vs.** | **Sham vs. OVX** | | **Sham-Veh vs.** |
| --- | --- | --- | --- | --- | --- | --- | --- | --- | --- | --- | --- | --- | --- | --- |
| **Infusion group:** | | **Vehicle** | | **HTN** | | **HTN** | **Vehicle** | | **HTN** | | **HTN** | **Veh** | **HTN** | **OVX-HTN** |
| **Property** ^a^ | **Units** | Mean | SD | Mean | SD | p-value | Mean | SD | Mean | SD | p-value | p-value | p-value | p-value |
| *Distal femur metaphysis* | | n = 11 | | n = 11 | |  | n = 11 | | n = 13 | |  |  |  |  |
| Tb.N | 1/mm | 1.56 | 0.19 | 1.64 | 0.10 | NA | 1.61 | 0.24 | 1.50 | 0.20 | NA | NA | NA | NA |
| Tb.Sp | µm | 657 | 106 | 618 | 38 | NA | 637 | 85 | 683 | 91 | NA | NA | NA | NA |
| Conn.D | 1/mm^3^ | 4.34 | 2.19 | 7.30 | 7.26 | NA | 7.26 | 9.99 | 9.87 | 7.13 | NA | NA | NA | NA |
| Tb.TMD | mgHA/cm^3^ | 899 | 23 | 913 | 28 | 0.4545 | 887 | 35 | 849 | 24 | 0.0360 ^c^ | 0.5032 | <0.0001 ^c^ | 0.0009 ^c^ |
| SMI | 0:plates; 3:rods;  4:spheres | 3.3 | 0.4 | 3.2 | 0.5 | NA | 3.4 | 0.4 | 3.0 | 0.5 | NA | NA | NA | NA |
| *L6 vertebral body* | | n = 12 | | n = 11 | |  | n = 12 | | n = 13 | |  |  |  |  |
| Tb.N | 1/mm | 2.92 | 0.26 | 2.77 | 0.42 | NA | 2.74 | 0.43 | 2.59 | 0.47 | NA | NA | NA | NA |
| Tb.Sp | µm | 357 | 33 | 385 | 56 | NA | 382 | 62 | 408 | 66 | NA | NA | NA | NA |
| Conn.D | 1/mm^3^ | 57.0 | 13.5 | 74.3 | 21.8 | NA | 70.3 | 26.1 | 75.6 | 30.3 | NA | NA | NA | NA |
| SMI | 0:plates; 3:rods;  4:spheres | 1.1 | 0.4 | 1.1 | 0.3 | NA | 1.4 | 0.4 | 1.6 | 0.7 | NA | 0.1385 | 0.0199 | NA |
| Bone Area | mm^-3^ | 0.57 | 0.08 | 0.56 | 0.07 | NA | 0.52 | 0.10 | 0.47 | 0.14 | NA | 0.2498 | 0.0741 | NA |
| EFL ^d^ | N | 16.8 | 2.4 | 13.6 | 3.2 | 0.0881 ^c^ | 12.8 | 3.5 | 10.0 | 4.5 | 0.0654 | 0.0204 | 0.0182 | NA |

^a^ Properties come from μCT evaluations of the distal femur metaphysis or 6^th^ lumbar vertebral body; ^b^ Not applicable (NA) because the fixed effect in the ANOVA was not significant or because the interaction between surgery and infusion was not significant (Table 2); ^c^ Adjusted P-values come from Dunn’s pairwise comparison because group values violated normality assumption or homoscedastic assumption. ^d^ Estimated Failure Load (EFL) from finite element analysis.


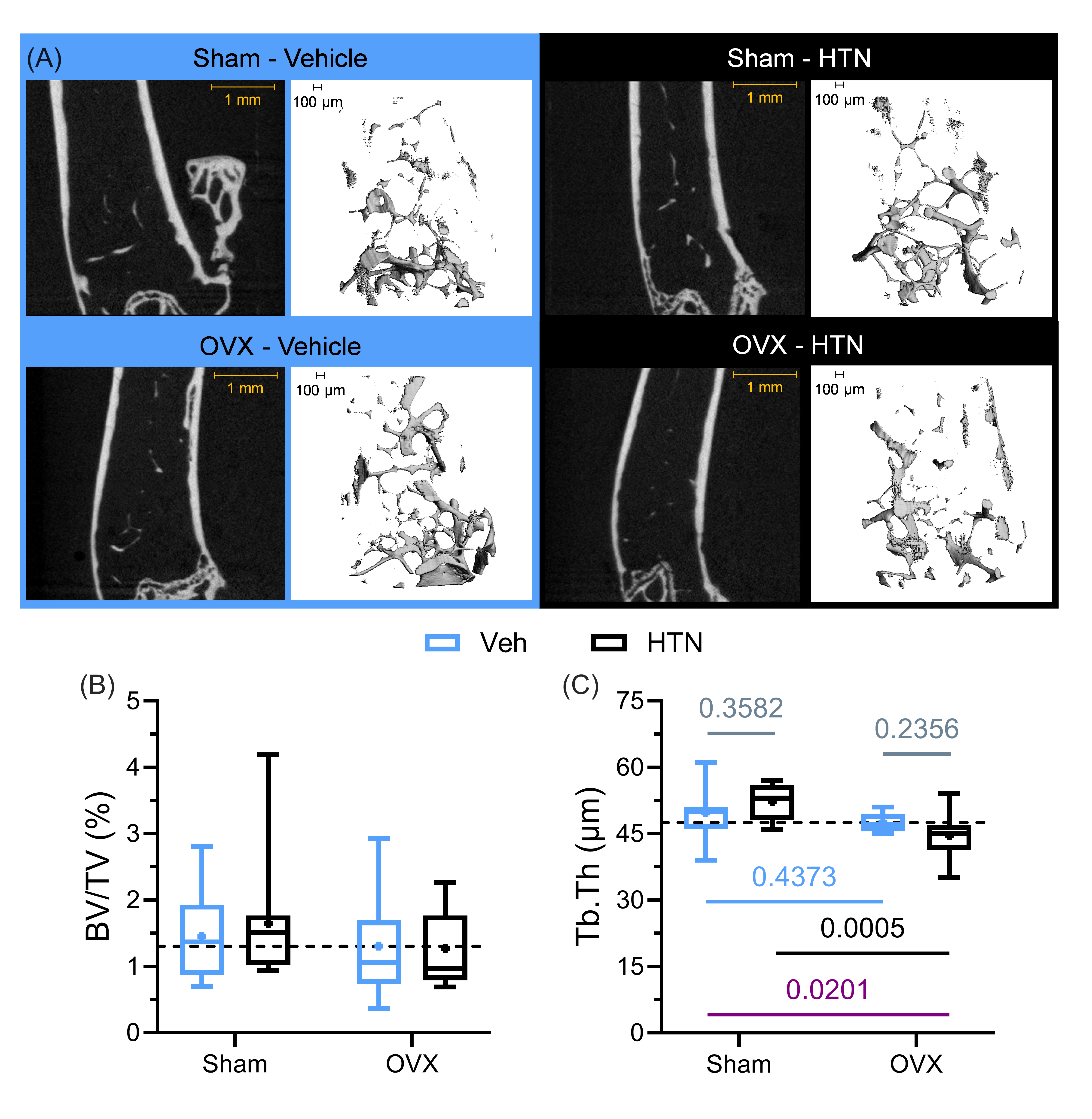


**Figure S1. Trabecular bone volume fraction and architecture in the distal femur metaphysis.** Regardless of group, there was relatively little trabecular bone in the aged female (>14 months of age) mice (A). Perhaps because of this, neither OVX nor HTN affected trabecular bone volume fraction (B). Nonetheless, OVX reduced trabecular thickness but only when the mice were hypertensive (C). ANOVA p-values reported in Table 2 for all parameters. Additional μCT parameters can be found in Table S1.
